# Nanoparticle-mediated cancer cell therapy: basic science to clinical applications

**DOI:** 10.1007/s10555-023-10086-2

**Published:** 2023-02-24

**Authors:** Jaya Verma, Caaisha Warsame, Rajkumar Kottayasamy Seenivasagam, Nirmal Kumar Katiyar, Eiman Aleem, Saurav Goel

**Affiliations:** 1 School of Engineering, London South Bank University, London, SE10AA UK; 2 Surgical Oncology, PSGIMSR, Coimbatore, India; 3 School of Applied Sciences, Division of Human Sciences, Cancer Biology and Therapy Research Group, London South Bank University, London, SE10AA UK; 4 Department of Mechanical Engineering, University of Petroleum and Energy Studies, Dehradun, 248007 India

**Keywords:** Nanoparticles, Cancer, HEAs, MD simulations

## Abstract

Every sixth person in the world dies due to cancer, making it the second leading severe cause of death after cardiovascular diseases. According to WHO, cancer claimed nearly 10 million deaths in 2020. The most common types of cancers reported have been breast (lung, colon and rectum, prostate cases), skin (non-melanoma) and stomach. In addition to surgery, the most widely used traditional types of anti-cancer treatment are radio- and chemotherapy. However, these do not distinguish between normal and malignant cells. Additional treatment methods have evolved over time for early detection and targeted therapy of cancer. However, each method has its limitations and the associated treatment costs are quite high with adverse effects on the quality of life of patients. Use of individual atoms or a cluster of atoms (nanoparticles) can cause a paradigm shift by virtue of providing point of sight sensing and diagnosis of cancer. Nanoparticles (1–100 nm in size) are 1000 times smaller in size than the human cell and endowed with safer relocation capability to attack mechanically and chemically at a precise location which is one avenue that can be used to destroy cancer cells precisely. This review summarises the extant understanding and the work done in this area to pave the way for physicians to accelerate the use of hybrid mode of treatments by leveraging the use of various nanoparticles.

## Introduction

In 2020, 19.3 million new cases of cancer were reported and nearly 50% of this succumbed to death. According to the American cancer society, it is expected that the number will grow to about 28.4 million new cancer cases and 16.3 million cancer deaths by 2040 which will be a 47% rise from 2020 [1]. It is noteworthy to mention that in 1927, cancer was named one of the top three causes of death in America by the US Census Bureau (*AACR: Landmarks in cancer research. 1907–2007*). Over these 100 years, although therapeutic advances have increased the overall survival (OS) rates, there is still no cure for cancer. Some of the earliest evidence of cancer suggestive of osteosarcoma was found in human mummies in ancient Egypt. The oldest description of cancer from the so called the ‘Edwin Smith Papyrus’ dates to about 3000 BC in Egypt. The papyrus, which is from an ancient trauma surgery textbook, describes 8 cases of breast tumours or ulcers that were removed by cauterisation with a tool called fire drill. Hippocrates (460‐360 BC) is attributed with giving tumours the name Karkinoma ‘carcinoma’, and thus ‘cancer’ from the finger-like extensions (veins) stemming from the main body of a breast lesion that resembled a crab [2]. Over the last 2000 years, progress in our knowledge about cancer was slow until the twentieth century when the discovery of DNA double helix in 1953 by Watson and Crick, and later the human genome project revolutionised our knowledge of genomes, and cancer biology.

**Fig. 1:**
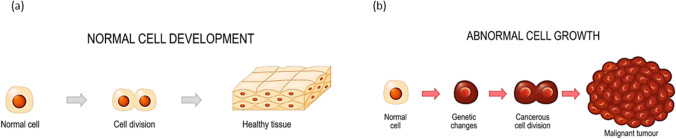
Image illustrating the cell development showing **a** normal cell growth and **b** abnormal cell growth [4]

The cancer cell has a set of eight hallmarks or functional capabilities characteristic of malignancy, which include uncontrolled cell proliferation, and evading apoptosis [3]. The process of normal and abnormal cell growths can better be understood from Fig. 1, which shows how a normal tissue transforms into a tumour causing death of individuals.

There are two types of tumours (i) benign and (ii) malignant (cancerous). Currently, more than 100 different types of cancer exist and are named after either the tissue or organ they originate from.

Cancers that starts in specific kinds of cells fall into the following categories: carcinoma is the most frequent cancer that arises in epithelial tissue and affects the breast, kidney, liver, skin, lungs gland pancreas, neck and head [5]. Sarcoma affects connective tissues including muscles, bones, blood vessels and cartilage. Leukaemia arises mainly from the bone marrow. Myeloma and lymphoma are malignancies of the immune system [6]. Other types of tumours include Germ cell tumours and neuroendocrine tumours and carcinoid tumours, which are generally detected in the gastrointestinal system [6] [7]. Additionally, one vital feature of cancer cell is the unusual growth from a single cell, usually harbouring driver mutation (s) that result in clonal expansion. Such development was observed by the analysis of X chromosome inactivation which occurs randomly during embryonic proliferation and are the single cell origin of various tumours [8]. The multistep process of cancer development, at the cellular level, involves genetic and epigenetic modifications that drive cellular transformation and the acquirement of sustained proliferation survival, invasion and metastasis [7, 8]. Treatment of benign tumour involves surgical removal if it compresses nearby structures or becomes gradually malignant. Treatment of malignant tumours consists of multi-modality treatment involving combinations of radiotherapy, immunotherapy, surgery, chemotherapy or targeted therapy.

R&D labs across the globe have focussed their research on early detection and cost effective treatment of various cancers [9]. Over the last decade, the treatment of cancer is moving towards precision medicine and less invasive strategies. Targeted therapy of cancer involves molecular level manipulation of proliferation, angiogenesis, cell death, invasion or immunosuppression. The rapid advances in nanotechnology enables the integration of inorganic nanomaterials with biomatter and drugs, which can be used for early detection, and treatment of cancer [10]. Nanoscience has opened new vistas to treat chemoresistant cancer cells through targeted delivery [11, 12]. Several nanoscience integrated therapeutics have evolved from basic research to clinical discoveries [12]. Different kinds of nanoparticle technologies like low frequency mechanical vibrations by magnetic nanoparticles are being used to accelerate cell death [13].

The treatment of cancer cells using a materials science approach presents a great opportunity for multidisciplinary research. The targeted use of nanoparticles can enable new treatment methods. Nanoparticles are 1–100 nm size atomic clusters of matter. Research into inorganic nanoparticles and their interactions with the biological systems are in developing stages to establish their response and tune them as per desired properties by functionalisation [14] as well as nanocarriers [12]. Due to their high surface-to-volume ratio, high density of binding sites can be accommodated and triggered for their function such as binding to specific sites, release the drug at certain time/temperature/pH, in controlled manner, etc.

## Historical perspective of cancer treatment

There are several approaches towards cancer treatment including surgery, radio- and chemotherapy, and most recently targeted immunotherapy.

### Surgery

Historically, Maimonides in AD 1190 appears to be the first to document surgery as a method to remove tumours [15]. It was not until the nineteenth and early twentieth centuries that major advances were made in cancer surgery, especially after anaesthesia became available in 1846. Halsted developed the radical mastectomy during the last decade of the nineteenth century [16]. Most women with breast cancer nowadays have the primary tumour removed followed by adjuvant therapy that may include radiation, chemotherapy, targeted, or hormonal therapy. Progress in cellular, molecular and imaging techniques in the twentieth century was instrumental in the advancement of surgical techniques. For example, ultrasound (sonography), computed tomography (CT scans), magnetic resonance imaging (MRI scans) and positron emission tomography (PET scans) have replaced the exploratory surgeries used previously to diagnose cancer. Advances in surgical techniques also include laparoscopic, thoracoscopic surgeries, endoscopy, lasers, cryoablation and radiofrequency ablation [17].

The discovery and understanding of metastasis placed limitations on the use of surgery to treat invasive cancer, and other therapeutic interventions have been introduced.

### Radiotherapy

Shortly after the discovery of X-rays by Wilhelm Rontgen in 1895, radiotherapy as a treatment option for cancer started to emerge. Currently, almost half of all patients with cancer are treated with radiation. Ionising radiation induces DNA damage leading to cancer cell death, but it has toxic effects on normal tissue [18]. Few years after its discovery, radiation therapy was found to cause cancer. Radiation carcinogenesis was established in human populations, and the dose–response relationship was described in radiation leukaemia [19]. Advances in radiation physics and computer technology in the twentieth century made it possible to aim radiation more precisely at tumours. For example, conformal radiation therapy (CRT) allows three-dimensional anatomical information of the tumour and surrounding healthy tissues, thus facilitating the establishment of three-dimensional conformal radiotherapy (3D-CRT) and radiation beams are delivered to the tumour from several directions. Intensity-modulated radiation therapy (IMRT) allows the intensity of the beams to be adjusted, thus delivering high dose to the cancer while decreasing the dose reaching the surrounding normal tissue [20]. Several technologies have been developed, the goal of which is to protect the surrounding normal tissue from the DNA damaging effects of radiation.

### Chemotherapy

The discovery of chemotherapy was a result of observations that soldiers in World War II exposed to nitrogen mustard had low white blood cells count. This led to the discovery that intravenous nitrogen mustard slowed the growth of lymphomas and leukaemia in patients refractory to radiotherapy [21]. Nitrogen mustard was approved for cancer treatment in 1949. Later in 1948, and in 1950, the first successful chemotherapy for childhood leukaemia, and the first rationally conceived nucleotide analogue chemotherapeutic agents were developed, respectively [22, 23]. Aminopterin discovered by Farber was the predecessor of methotrexate, which is commonly used today. Since then, hundreds of chemotherapeutic agents were developed and proved successful in inducing long-term remission. The use of combination chemotherapy proved advantageous over single agents. Some types of very fast-growing leukaemia and lymphoma respond very well to combination chemotherapy. For example, the remission induction therapy for acute myeloid leukaemia (AML) consists of 4–5 cycles of intensive chemotherapy, which typically includes cytarabine (Ara-C), the backbone for many therapeutic regimens, combined with etoposide and anthracycline [24]. Standard treatment with intensive induction chemotherapy for AML induces complete remission (CR) in 60–80% of patients aged 60 years and under. In paediatric AML, the overall remission-induction rates are approximately 85 to 95% and event-free survival (EFS) rates range from 50 to 65% [25].

Cytotoxic chemotherapy has many limitations such as drugs cannot distinguish between normal and cancer cells, they cause significant side effects that affect the quality of life of patients, and they usually target fast proliferating cancer cells, not cancer stem cells. For example, following 5-day cultivation with gemcitabine *in vivo*, pancreatic cancer stem cells were enriched up to 47.2% compared to 1.47% in a primary cancer cell line [26]. One of the major challenges that affects patients’ response to chemotherapy is drug resistance. For example, acquired resistance to Ara-C is a major obstacle in the clinical management of AML. Increasing the intensity of the current chemotherapy regimens does not improve outcomes because of the high percentage of treatment-related deaths (5–10%), and of long-term side effects [27]. Repurposing drugs such as disulfiram has been useful in overcoming Ara-C and bortezomib resistance in Down syndrome–associated AML cell lines [28].

### Targeted therapy

The advances in our understanding of cancer biology and the human genome revolutionised therapeutics which block specific molecular pathways essential for cancer cell survival, cancer growth, progression and metastasis. Targeted cancer therapies are developed to interrupt a specific component of the complex network of altered signalling pathways that ultimately results in uncontrolled cell proliferation [29]. Molecular targeted therapies have shown remarkable success in the treatment of several cancer types including breast, leukaemia, colorectal, lung and ovarian cancers [30]. The first targeted cancer therapy was tamoxifen approved in 1977 for the treatment of breast cancer. Tamoxifen binds to the oestrogen receptor (ER), and therefore modulates ER activity, thus providing an effective treatment option for patients with ER-positive breast cancer [31]. Several mechanisms lead to aberrant functions by protein tyrosine kinases encoded by oncogenes, and subsequent cellular transformation. These include genomic rearrangements resulting in oncogenic fusion proteins (examples include BCR-ABL in chronic myelogenous leukaemia, PAX3-FOXO1 in alveolar rhabdomyosarcoma). Additionally, gain-of-function mutations, overexpression, gene amplification, and loss of the normal regulatory constraints of kinase activation [31]. Growth factor antagonists and growth factor receptor inhibitors are used as effective targeted therapeutic approaches to suppress progression and metastasis of cancer cells and sensitise the cells to killing by cytotoxic anticancer agents. Examples include Her2 antibodies targeting the Her2 receptor in breast cancer, and small molecule inhibitors targeting EGFR, IGF-1R, VEGFR and PDGFR [32].

One limitation of targeted therapy is drug resistance. There is a significant cross-talk between receptor tyrosine kinase pathways, and the same pathway is usually activated by multiple receptors. Therefore, if the function of one protein located upstream the signaling pathway is inhibited, another protein will most likely compensate the interrupted function. The outcome will still be uncontrolled proliferation [29].

Targeted immunotherapy has proven successful in many types of cancer. It harnesses the immune system to attack cancer cells. Cancer immunotherapy includes monoclonal antibodies, cancer vaccines, immune checkpoint inhibitors, CAR T cell therapy and immune system modulators. Limitations of cancer immunotherapy include resistance, escape of cancer cells from the immune response and issues related to delivery methods. Some of these issues could be resolved by using nanocarriers as vehicles because of their increased surface areas, targeted delivery, controlled surface and release chemistry, enhanced permeation and retention effect [33].

## Nanoparticle advantages in cancer therapy

Nanoparticles (1–100 nm) can be used to treat cancer because of their unique characteristics like biocompatibility, reduced toxicity, increased permeability, improved stability, precision targeting and retention effect. Magnetic nanoparticles in particular offer several appealing features for biomedical applications [34, 35]. They have so far been extensively researched and used for magnetic resonance imaging (MRI) as contrasting agents [36, 37] particularly for drug delivery [38–40] and occasionally in conjunction with MRI [41], as part of the theragnostic concept, cell sorting, regenerative medicine [42], tissue engineering [43, 44] for hyperthermia [45–47] and for protein purification [48]. An external magnetic field can be used to remotely control magnetic nanoparticles, which are nanometric-sized compounds with unique magnetic characteristics. Due to their high surface-to-volume ratio, it is possible to graft many molecules onto their surface. Additionally, it encourages their contact with living cells, proteins, viruses and DNA [13, 49].

### Magneto-mechanical effect

**Fig. 2:**
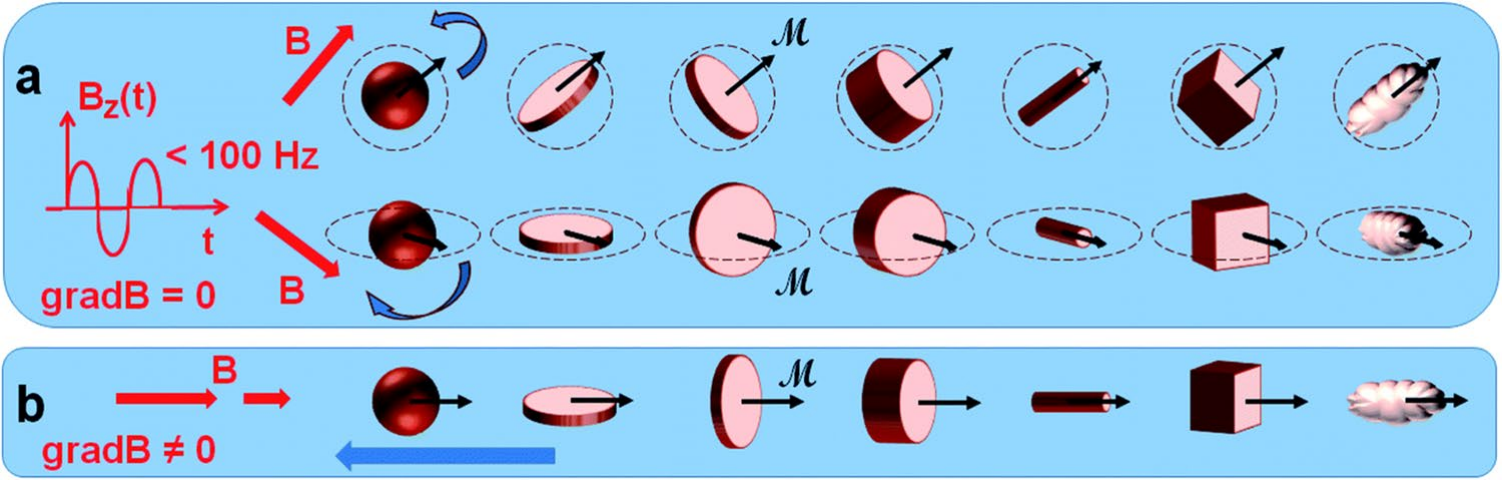
Schematic of numerous types of magnetic particles exposed to variable magnetic fields, thus subjected to magneto-mechanical torques, tending to rotate the particles [13]

A newly developing area of research is the therapy of cancer using the magneto-mechanical effect of particles (TMMEP) [34]. The basic idea behind this method is to exert mechanical pressure on cancer cells in order to cause magnetic particle vibrations, which will ultimately cause the cell death [13, 50]. For this, a low-frequency alternating magnetic field is used as shown in Fig. 2 and magnetic particles are injected into the tumour or exposed to cancer cells. The average magnetic moment *M* of the particle, which depends on the amplitude and direction of the applied field *B*, is subjected to the magnetic torque *M* × *B* and tends to align with the direction of the field in an applied magnetic field *B* that is assumed to be uniform over the entire volume of the particle. Meanwhile, if the magnetic anisotropy of the particle is high enough (exhibiting strong particle-composition, size and shape dependence), the direction of the magnetic moment *M* remains almost blocked within the particle, parallel—or making a small angle—with the axis known as the easy axis of magnetisation, or maintained in the easy plane of magnetisation. The action of the magnetic torque becomes magneto-mechanical on particles separated from fluidic solutions or that are only partially attached. Similar to how Earth’s magnetic field affects a compass needle, it tends to reorient the particle until its easy axis or easy plane align with the applied magnetic field direction. Thus, the particles in TMMEP are continuously rotated or vibrated using rotating, or more generally changing, spatially homogeneous magnetic fields. Highly anisotropic particles, such as magnetic discs with ‘magnetic shape anisotropy’ or with ‘perpendicular magnetic anisotropy’, are usually recommended in this method demanding effective magnetic torque [51].

### Organic–inorganic interaction (hybridisation/functionalisation)

The primary determinants of the biocompatibility and uptake effectiveness of the nanoparticles used for cancer therapy are thought to be the physicochemical characteristics of NPs, including surface composition, size, superficial charge and shape. Through the functionalisation of the NP surface, it is feasible to increase the biocompatibility and uptake effectiveness of NPs by altering their physicochemical properties. Theragnostic properties of functionalised nanoparticles have completely transformed the way in which cancer is treated. The method of surface functionalising NPs attempts to enhance and/or add features beneficial for using NPs in medicinal applications. Different kinds of nanomaterials have distinctive chemical characteristics and functional groups that can be exploited in the initial stages of functionalisation since they are accessible on their surfaces [52]. Typically, the initial stage of surface modification involves adding an organic functional group (R-NH_2_, R-COOH, etc.) that can be used to bind biological molecules using homo- or hetero-bifunctional cross linkers. The most popular linkers for silica NPs are aminosilanes, which adds an amino group to the NPs’ surface in preparation for the upcoming bioconjugation. Crosslinkers containing -SH or -NH_2_ groups can be used to functionalise noble metals, such as gold, by reacting with the metal and creating a covalent bond [53].

These bifunctional linkers, like thio-carboxylic acids have functional groups at the other end that can be used to bind ligands [54]. By replacing the original surfaces of metal oxides with functional groups like diol, amine, carboxylic acid and thiol useful for the following processes, metal oxides can be easily modified [55]. The *sp*^*2*^ hybridised carbon atoms present in considerable amounts in the carbon-based nanomaterials can be used to produce functional groups. It is possible to produce -COOH, -OH and -C = O on the surface of NPs through oxidation that can be further modified, for instance by reaction with the amine group and cycloaddition can introduce various types of functional groups. Table 1 lists various types of nanomaterials, their chemical compositions and/or groups, and the appropriate substances or methods that can be employed for surface modification utilising crosslinkers [52].

**Table 1:** A list of the most popular methods for modifying the surface of NPs [52]

Material	Usable functional/chemical groups	Example of chemical compounds/processes suitable for surface modification
Silica	-SiOH	X-Si (OC_2_H_5_)_3_
Noble metals	-Au; -Ag (plasmonic metals)	X-SH, X-NH_2_
Metal Oxide	MO_x_	X-COOH; X-(OH)n;X-NH_2_
Carbon based	Sp^2^ hybridise carbon	Oxidation; halogenation; cycloaddition

Non-covalent conjugation and covalent conjugation are two alternative methods that can be used to modify the surface of NPs.

The non-covalent approach, which is specifically utilised with metallic and silica NPs is based on a variety of weak interactions, including electrostatic, ionic, van der Walls and hydrophobic contacts, absorption and hydrogen bonding [56]. Non-covalent bonds have the benefit of being quite straightforward and unaffected by the structure of the molecules being utilised or how they interact with biological targets. However, various factors like pH and ionic strength can quickly affect non-covalent transformations. Depending on the nature of the NPs, various other methods can be used to create the covalent bonding mechanisms [57]. Additionally, utilising successive functionalisation, this approach enables alterations at many levels [58]. In order to execute the theragnostic approach, this methodology can be used to create structures with various purposes, such as diagnostic and therapy. Usually, different linker molecules can be used to covalently attach ligands to the surface of NPs. PEG is a prime example. It can be synthesised with certain functional groups at the ends and utilised as homo-bifunctional or hetero-bifunctional linkers to carry out a variety of functionalisation activities.

**Fig. 3:**
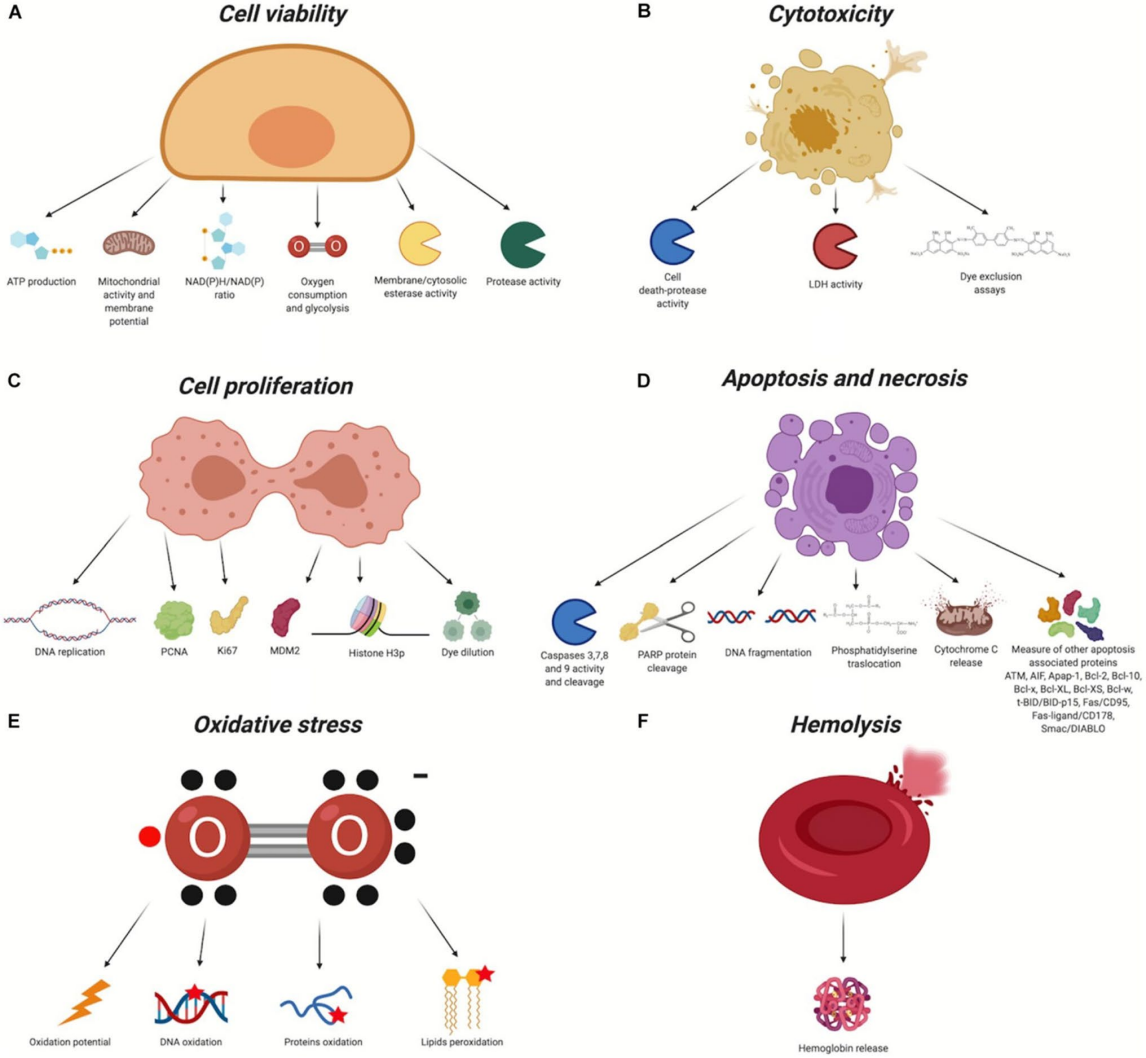
Biocompatibility evaluation assays. In red, the chemical groups that react with the nanomaterial. X, organic/inorganic free chemical groups used to bind the ligands. **A** Cell viability. **B** Cytotoxicity. **C** Cell proliferation. **D** Apoptosis and necrosis. **E** Oxidative stress. **F** Haemolysis [52]

The large number of scholarly works devoted to this subject attest to the effectiveness of NP surface modification in enhancing uptake and biocompatibility. Due to the modification of surface charge and the inactivation of reactive chemical groups that may impact cellular membrane stability, these findings show that conjugating molecules on the surface of NPs can effectively improve biocompatibility both in vivo and in vitro (Fig. 3). Additionally, the inclusion of certain molecules can improve the passive and active uptake of NPs, minimising in vivo systemic toxicity and enabling very precise therapy and/or diagnosis. Both covalent and non-covalent methods can be used to bind compounds to the NP surface. In order to improve uptake and perform active targeting, the former is usually applied to bind proteins, aptamers, antibodies, and peptides, while non-covalent interactions are typically used for drug loading and for all molecules that must be released in cells [52].

### Clinical relevance

The benefits of functionalised nanocarriers, such as their enhanced permeation and retention, passive targeting capacity, capacity to load drugs for targeting modification, and high surface-to-volume ratio, made it possible to conduct a number of clinical research studies focusing on combined therapy [59]. For instance, Katragadda et al. [60] showed a safe and effective nanosized formulation for the delivery of paclitaxel and 17-AAG combination therapy, which has only yielded modest results in phase 1 clinical studies. Novel nanoparticles based on polymeric microspheres loaded with two anticancer medications were created by Liu et al. [61] for pulmonary transport. Studies on *in vivo* pharmacokinetics and biodistribution of the microspheres revealed that they had a long circulation time and might develop in the lung.

Araujo et al. [62] (Table 2) described tyrosine kinase inhibitors used in clinical practice for treating solid tumours. SRC is a tyrosine kinase that plays a crucial role in the oncogenic and bone-metastatic processes, making it a viable therapeutic agent for the treatment of solid tumours. One of the SRC inhibitors now under development and the current findings are helpful in determining if targeting SRC is an effective therapeutic approach. Experiments conducted *in vivo* and *in vitro* revealed that the NPs exhibit superior antitumour effect and lower toxicity [63, 64].

**Table 2:** Tyrosine kinase inhibitors used in clinical trials for treating solid tumours

Tyrosine kinase inhibitor	Kinase target(s)	FDA-approved indications
Imatinib (Gleevec/Glivec)	BCR-ABL, c-KIT, PDGFR	CML, Ph + ALL, GIST
Dasatinib (Sprycel)	SRC, SFKs, BCR-ABL, c-KIT, PDGFR, c-FMS, EPHA2	CML (2nd-line), Ph + ALL
Gefitinib (Iressa)	EGFR	NSCLC
Erlotinib (Tarceva)	EGFR	NSCLC
Nilotinib (Tasigna)	BCR-ABL, c-KIT, PDGFR	CML (2nd-line)
Lapatinib (Tykerb)	EGFR, HER2/neu	Advanced breast cancer
Sunitinib (Sutent)	VEGFR2, PDGFR, c-KIT, FLT3	GIST, renal cell carcinoma
Sorafenib (Nexavar)	VEGFR, PDGFR	Renal cell carcinoma, hepatocellular carcinoma

*EGFR* epidermal growth factor receptor, *CML* chronic myeloid leukaemia, *EPHA* ephrin A, *GIST* gastrointestinal stromal tumours, *FLT3* FMS-like tyrosine kinase 3, *NSCLC* non-small cell lung carcinoma, *Ph* + *ALL* Philadelphia chromosome–positive acute lymphoblastic leukaemia, *PDGFR* platelet-derived growth factor receptor, *VEGFR2* vascular endothelial growth factor receptor-2.

### Modelling and simulation studies

It is known that NPs have potential to ameliorate cancer treatment through to their highly developed functionalisation and their ability to accumulate in specific tumours. Yet, one common issue is the gap of knowledge on understanding the impact of NP designs (surface functionalisation, shape and size) to reduce the complication on transport barriers in the body [65]. Increased computational modelling methods, along with advanced multiscale simulations of NPs, tumours, and the biological transport barriers that influence them, permits us to explore the influence of a variety of designs in biologically related scenarios [66]. The development of effective NP cancer therapies can be accelerated with the use of in silico models [66] discussed further.

#### In silico* models of tumours*

Continuum, discrete and hybrid are the current mathematical methods used to model tumours. However, continuum models (which uses ordinary and partial differential equations [67] and are best suited to describe global changes to a tumour) are restricted in their ability to re-create cellular interactions, heterogeneity and other features which discrete modelling method perform best. With the discrete method, it can model realistic heterogenous development of tumour, it can provide critical insight on angiogenic vessel growth and/or the emergence of cellular resistance [66], by following the simple rules leading their lifespan and interaction with local environment and other agents. Both methods provide crucial information but due to their limitations, researchers have proposed to combine them to obtain the advantages of both, the hybrid methods, which develops on discrete models but merge them with gradients of variables, modelled using continuum equations [68].

**Fig. 4:**
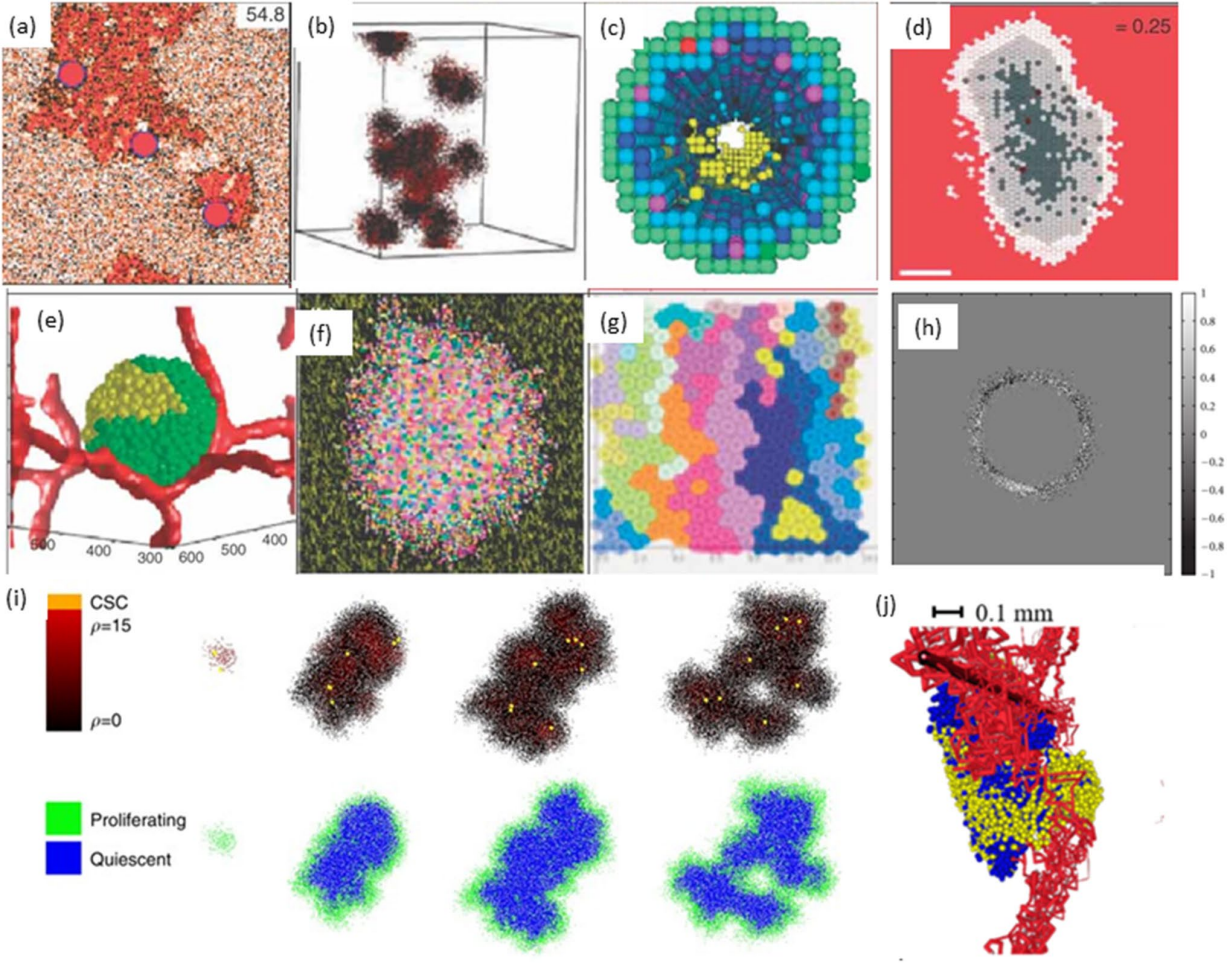
Schematic illustration of hybrid models of tumour growth developed by various researchers [73]. **a** In prostate ducts simulation of hybrid method of tumour invasion [74]. **b** Hybrid cellular method of 3D tumour self-metastatic [75]. **c** Square-grid cellular, *in situ* simulation of 3D model of ductal carcinoma [76]. **d** Hexagonal cellular mechanism of 2D spheroids tumour [77]. **e** Potts model simulation of 3D vascularised spheroid tumour [78]. **f** Potts model of a 2D spheroid tumour in a heterogeneous environment [79]. **g** Particle model with Voronoi triangulation simulation of 2D tumour [80]. **h** Simulation of the extracellular matrix in models of tumour growth [81]. **i** Simulation of cancer growth with multiscale agent-based modelling [82]. **j** Vascular tumour growth (blue: proliferating tumour cells, yellow: quiescent tumour cells) [83]

Various reviews are made on these topics which shows recent developments in hybrid models [69], discrete models [70] and multiscale modelling [71, 72] of cancer (Fig. 4).

#### Modelling of transport barriers for NP delivery

**Fig. 5:**
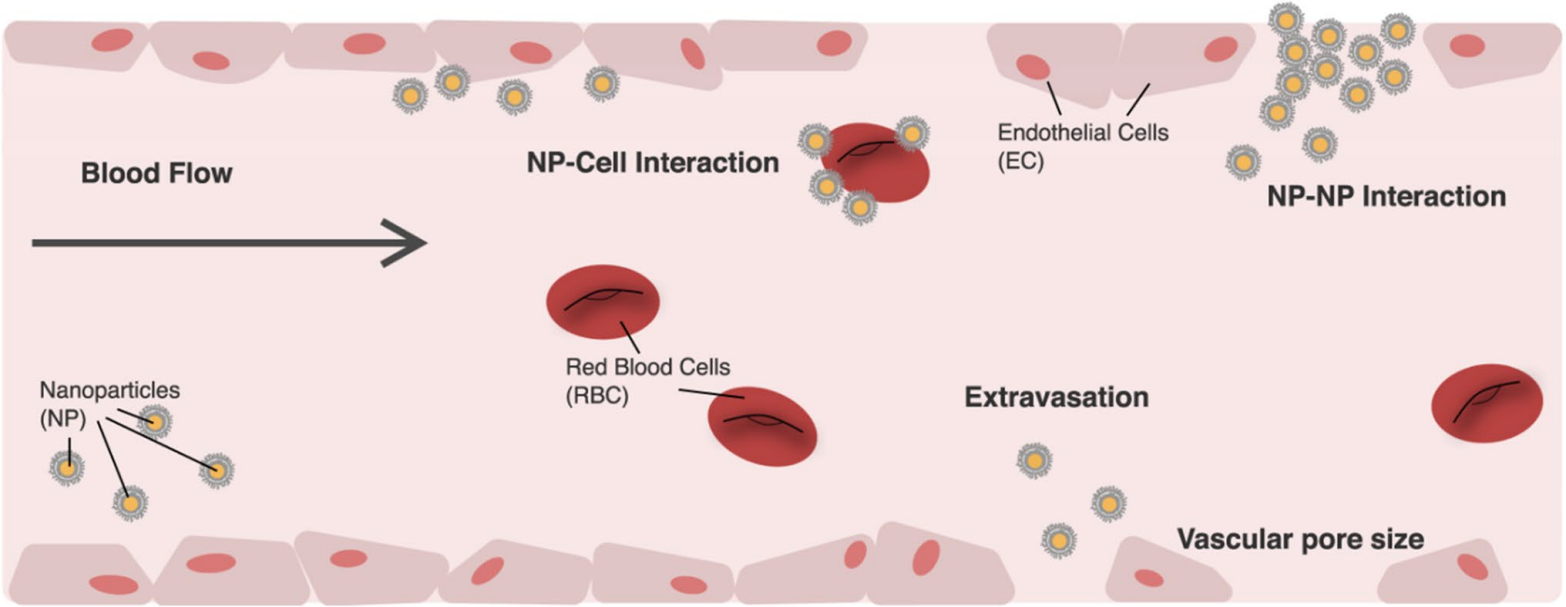
Image illustrating NP transportation across the vascular chamber. Consideration when modelling NPs [66]

To use NPs as a drug delivery pathway requires the particles to pass through from point of entry to their designated biological target in the body [66]. To overcome transport barriers, NPs require to travel through the vasculature [84, 85], extravasation [86], avoiding uptake by the reticulo-endothelial system [87], progression through the tumour tissue [88], endocytosis [89] and delivery to the relevant part of the cell [90], as shown in Fig. 5. It is known that when NPs are injected into the blood stream, there is a high probability for escaping into the tumour site through various arrival ports if the circulation time is prolonged [66]. These could be influenced by the design of the NPs including the charge, shape and size. When NPs are of size less than 5 nm, the kidneys rapidly clear them [91] and NPs greater than 100 nm have a high chance to be discovered and cleared by macrophages [92]. However, NPs between the two size will be transported by macrophage uptake [93].

**Fig. 6:**
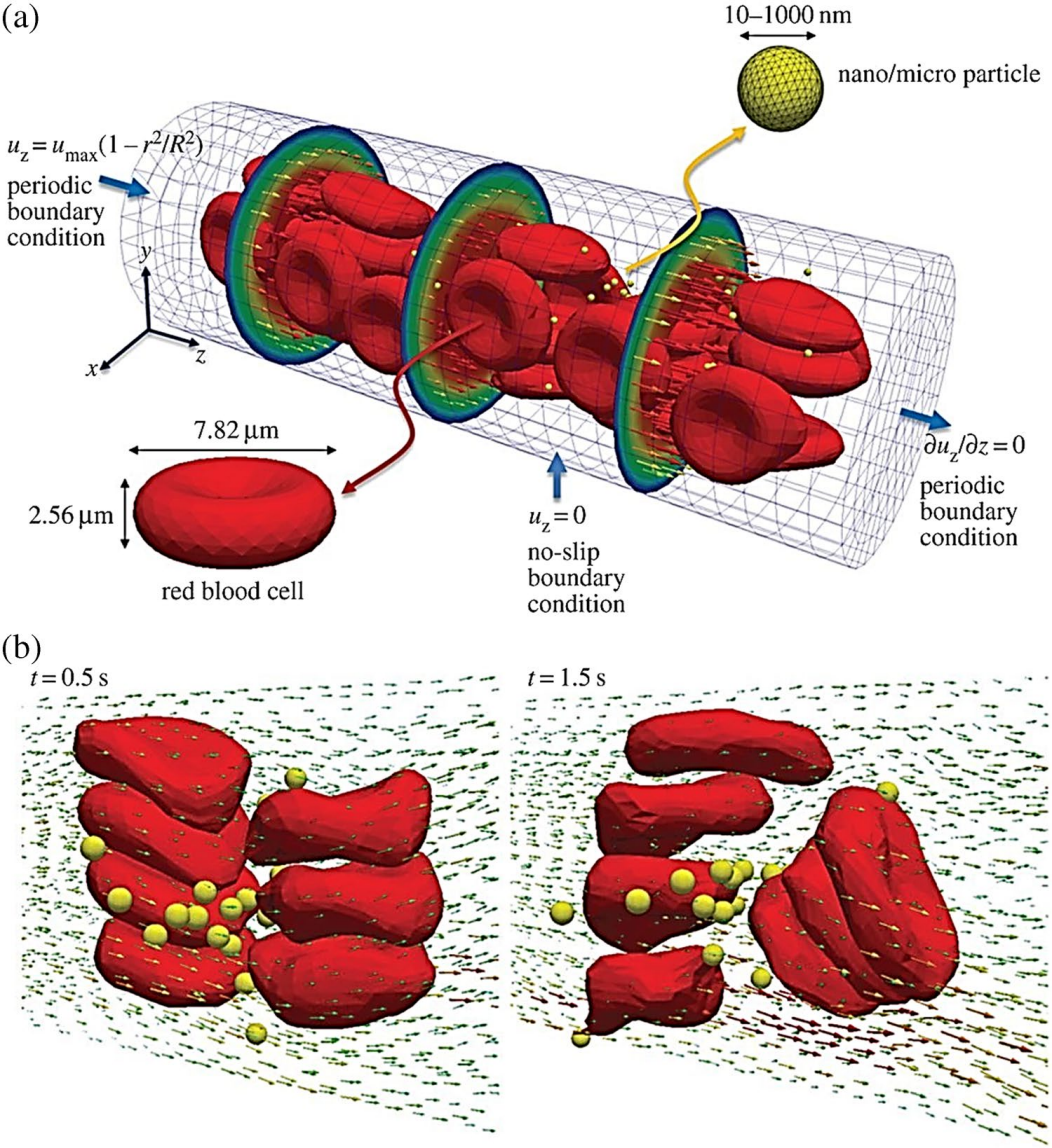
Image illustrating the microcirculation behaviour of the effect of NP shape and size. **a** Beginning structure of deformable red blood cells and spherical rigid particles scattered and **b** formation of complex flow field across the particles [65]

In the past, molecular dynamics (MD) simulations have been used to investigate the effect of NPs charge, shape, size, pH-stability and the protein corona formation influencing the NP transportation. For instance, Lopez et al. [94] used a coarse-grained model method to examine the adsorption effect of blood plasma proteins onto NPs. Shao et al. [95] used a coarse-grained approach to model the protein adsorption on NPs and simulated it using discontinuous molecular dynamics simulations. Maleki et al. [96] used single-walled carbon nanotube and multi-walled carbon nanotubes to study the pH-sensitive loading/releasing of doxorubicin. Additionally, other factors influencing the effect of NP transportation across the vascular are the margination, as the NPs can easily evade from the porous tumour vessel to the blood cells by preventing their interactions, as shown in Fig. 5. This was demonstrated in the study by Müller et al. [97] by combining the use of computational fluid dynamics and dissipative particle dynamics (DPD) to observe the margination effect. It was examined that in the core region within the red blood cells, NPs smaller than 200 nm are entrapped, but when NPs are larger than 500 nm the margination effect can be exploited as shown in Fig. 6 [65].

#### Tissue penetration and NP internalisation

**Fig. 7:**
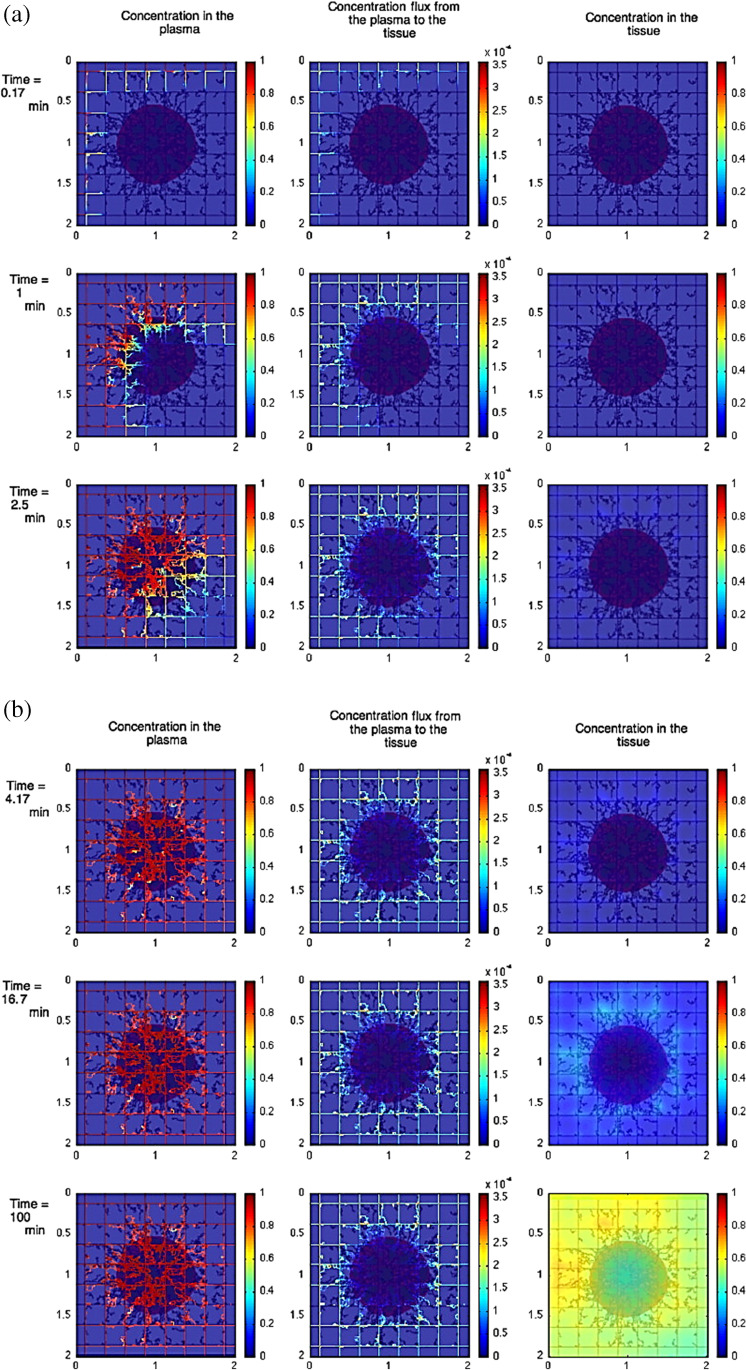
Image illustrating the effect of constant tumour tissue injection at the beginning vs later times. **a** The beginning time where red colour shows tumour with viable tissue, blue shows the hypoxic and brown shows the necrotic. **b** Later times, where left column shows the concentration in the plasma, the middle shows the concentration flux from plasma to the tissue, and the right column shows the concentration in the tissue [98]

One significant challenge for NP-based therapies is the prediction of NPs depth, their ability to penetrate into the tumour and the location of their accumulation [66]. With the use of in silico method such impediment can be better understood as it provides a mean of understanding the tumour penetration. For instance, Wu et al. [98] used a combination of agent-based modelling and fluid dynamics to investigate the influence of the interstitial fluid pressure on the lymphatic systems, the blood and their effect on drug delivery. This study showed the impact of increasing the drug distribution which resulted on a decrease respond on the lymphatic, see Fig. 7. For further investigation on the heterogeneous distribution of NPs, Wijeratne et al. [99] focused on investigating the conditions of the tumour for an improved NP drug delivery using a three-dimensional model continuum approach.

**Fig. 8:**
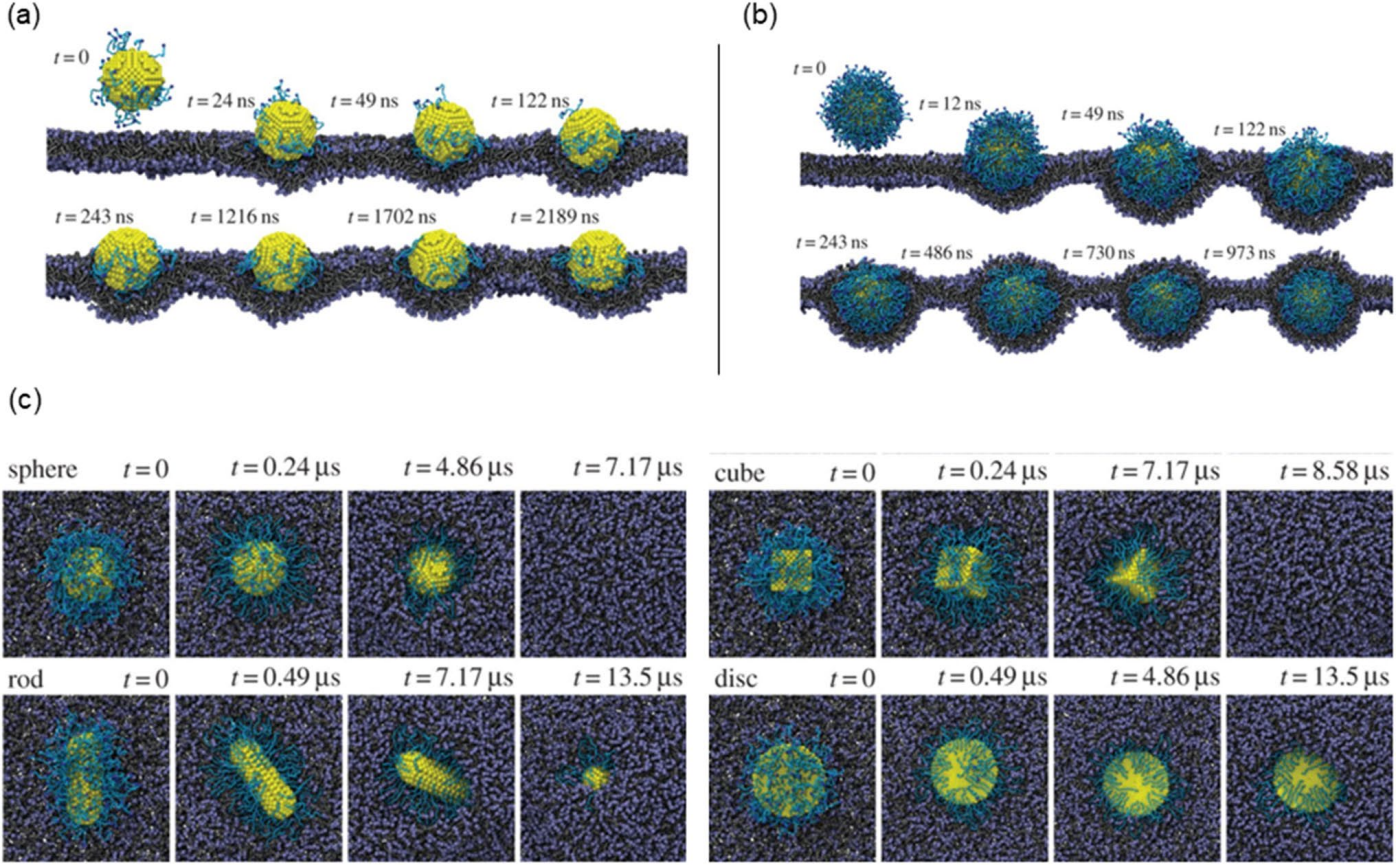
Image illustrating the NPs shape and surface functionality on the internalisation behaviour. **a** Different effects of PEGylated NP shape with grafting densities of 0.2. **b** Grafting densities of 1.6 chains nm.^−2^. **c** The effect of different shaped PEGylated NP internalisation impact process [65]

In terms of NP internalisation, Li et al. [65] presented early stages of receptor-driven endocytosis of NPs by introducing a novel dissipative particle dynamics technique. They examined the usage of multiscale modelling techniques to clarify how NP shape, surface functionalisation and size has an influence on their distribution by targeted cell in the microvasculature and following their internalisation. Quantitatively distinct behaviours of these PEGylated NPs were observed where the size of the PEGylated NPs with spherical core had a diameter of around 8 nm and a tethered chain with a molecular weight of around 838 Da. As presented in Fig. 8a, the PEGylated NP was initially absorbed on the surface of the cell membrane when the grafting density was 0.2 chains nm^−2^. After more than 2000 ns, the PEGylated NP was unable to penetrate the cell’s interior as it was still on the membrane’s surface. In contrast, as shown in Fig. 8b, when the grafting density was raised to 1.6 chains nm^−2^, the cell membrane began enfolding the PEGylated NP, followed by the membrane—extruding phase where NP was eventually completely encircled by the membrane and formed a complex of NP-membrane interactions. As such, to deliver the targeted drug, the PEGylated NP can be transported into the unhealthy cell.

As demonstrated in Fig. 8c, the NP models presented are spherical, rod-like, cubic and disc-like. Figure 8c shows that the shape of NPs plays a crucial role in designing the parameter for internalisation. The study conducted by Li et al. [65] compared different NP shape designs (first and second generation) that has similar surface areas for their cores and discovered that under a constant grafting density of 0.6 chains nm^−2^, the most efficient internalisation by the cell was observed to be as follows: the spherical, the cubic and lastly the rod-like NPs. However, it was observed that the disc-like NP was only detected on the cell membrane’s surface after being simulated at the same duration as the rod and resulted that it could not be ‘internalised’ as others. This emphasised that surface functionalisation plays another important role. Despite the fact that distinct NP shapes have various surface area to volume ratios, to distinguish the effect of NP size from the surface is challenging, particularly when little NP shapes are investigated. However, with accurate defined condition, there is a potential for an unambiguous investigation of these shapes that influences NPs during endocytosis, that is only possible through computer simulations [65, 100].

### Identification of the best NPs for targeted delivery

To maximise the mechanical reaction of the particles with the biological tissue, TMMEP characteristics such as structure, dimension and magnetic properties of the particles, frequency and amplitude of the applied magnetic field must be determined. For the particles to be utilised *in vivo* and subsequently for therapeutic purposes, the biocompatibility of the components that constitute the particles remains a key factor in their composition. Due to their biocompatibility, iron oxides like magnetite (Fe_3_O_4_) and maghemite (g-Fe_2_O_3_) are excellent choices, even though they often display non-negligible cytotoxicity [50, 101, 102]. According to the research by Ling [101] and Goiriena-Goikoetxea [50], bare iron oxide nanoparticles in particular are known to cause reactive oxygen species (ROS), which is thought to be one of the primary causes of nanotoxicity. Moreover, when iron oxide nanoparticles are covered in a biocompatible layer, based either on inorganic shells, like gold, silica, or tantalum coatings, or on a wide range of biocompatible organic shells, depending on the nanoparticle core type and the intended applications, their toxicity is significantly reduced. Alternatives that works well include magnetic materials made of nickel cobalt or NiFe alloys [13]. A gold coating or polyelectrolytes, for example, or limited dissolution should be ensured to maintain the biocompatibility of particles made of these hazardous metals. Surface functionalisation of particles are being required to precisely target a cell type or to improve particle dispersion in fluids [103, 104].

To achieve this, a layer of gold is deposited on the surface of the NPs, enabling the grafting of organic molecules via thiolates’ self-assembly on the gold surface. These thiolates frequently have functional terminal groups and a polyethylene glycol (PEG) spacer. The functional group enables the attachment of biomolecules for precise targeting while ensuring biocompatibility, while the PEG spacer promotes particle stability [105–107].

Magnetic particles are typically injected directly into the proposed area in vivo research [34, 37], preventing bloodstream absorption. Organising an injection of the particles via the venous route for prospective therapeutic applications is still challenging as long as the functionalisation strategies for delivering to the zone of interest are not efficient. The size and structure of the particles with the intervention of suitable functionalisation could be crucial for their circulation in the blood flow, at least close to the tumour site, even though venous injection remains difficult for targeting the tumour [39, 108]. Due to the phenomenon of flow margination, anisotropic shapes like nanodiscs, nanorods, and nanowires should be more advantageous than spherical ones. Additionally, if magnetically triggered by a different magnetic field, anisotropic particles enter the tumour site more effectively due to the EPR effect [13]. Some of the effective materials for these applications are discussed in the following sections:

(i) Iron oxide nanoparticles (IONPs)

**Fig. 9:**
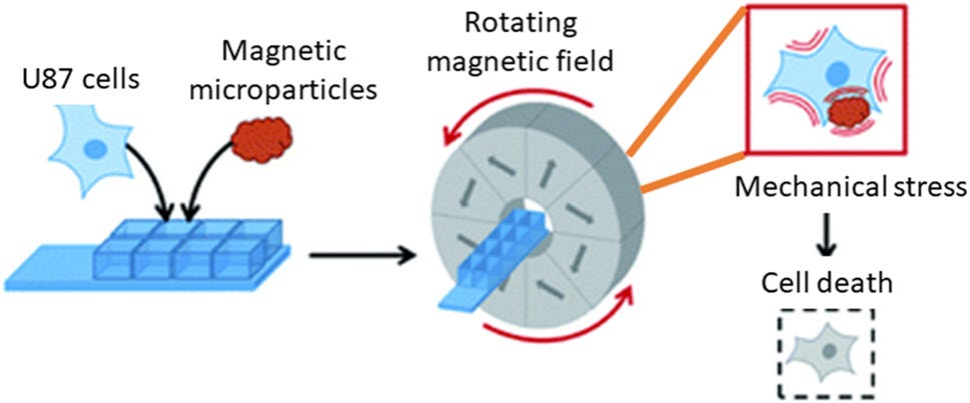
IONPs for anti-cancer therapy [116]

Latest reports have highlighted new IONP therapeutic potentials [109]. IONPs produce mechanical stresses and torques in response to low-frequency magnetic fields, which are transferred to the materials with which they come into contact. These torques can be utilised to modify molecules, improve gene transfection or tissue engineering, control calcium entry within cells, trigger protein degradation, activate enzymes or kill cancer cells (Fig. 9) [13, 110–114]. Sara et al. [115] demonstrated that pancreatic cancer-associated fibroblasts used as a model may be killed by a superparamagnetic iron oxide nanoparticle as small as 6 nm. An extensive analysis of magnetic field amplitude, frequency and type (rotating vs. alternating) revealed that the rotating low-amplitude low-frequency magnetic field (1 Hz and 40 mT) had the best effectiveness, reaching a 34% ratio in inducing cell death. Interestingly, cell death does not occur at the largest amplitudes of the magnetic field.

Modern kinetic Monte-Carlo calculations that could determine the torque experienced by magnetic nanoparticle assemblies explained these characteristics and agreed with cell killing investigations. Simulations revealed that the force the nanoparticles produced once they were ingested into the lysosome was about 3 pN, which is, in theory, insufficient to cause a direct membrane breach. Additional biological explanations for cell death have been investigated. Lysosome membrane permeabilisation and the release of lysosome content were caused by the mechanical activation of magnetic nanoparticles, and cell death was mediated by a lysosomal pathway that depended on cathepsin-B activity. By providing proof-of-concept that ultra-small nanoparticles can disrupt the tumour microenvironment through mechanical forces produced by the mechanical activation of magnetic nanoparticles upon exposure to a low-frequency rotating magnetic field, this study opened up new therapeutic possibilities for cancer (Fig. 9) [115].

(ii) Fe/Ni alloy

Kim et al. [117] used comparatively large nanoparticles with a 20/80% Fe/Ni alloy coated with gold in a disc geometry (60 nm thick and 1 m in diameter) in another study on cancer therapy. Human glioblastoma cell membranes were the target of functionalised anti-human-IL132R antibodies on the gold surfaces of the discs. They exposed the cells and particles to homogeneous AC magnetic fields with frequencies (10–20 Hz) and relatively low intensities (8 kA/m). The glioblastoma cells underwent apoptosis, and it was proposed that the discs aligned in the field and then slightly misaligned when the field was altered, harming the cell membranes to which they were linked and further resulting in an ionic signal that led to cell apoptosis [118].

(iii) Graphene quantum dots (GQDs) core–shell composite

In a study published by Fangjie et al. [119], a multimodal therapeutic system was demonstrated to be significantly more lethal in the destruction of cancer cells than a single dimension of nanotherapy, whether it be photothermal or photodynamic. Hollow magnetic nanospheres (HMNSs) were created to combine the benefits of photothermal and magnetomechanical cancer therapies. The cancer cells were structurally and physically eliminated by these combined stimuli, and their parameters were noticeably different from those caused by other therapies. As a core–shell composite, HMNS/SiO_2_/GQDs, the silica shells were also applied to HMNSs and coupled with carboxylated graphene quantum dots (GQDs). The composite was additionally stabilised using liposomes and loaded with the anticancer drug doxorubicin (DOX).

**Fig. 10:**
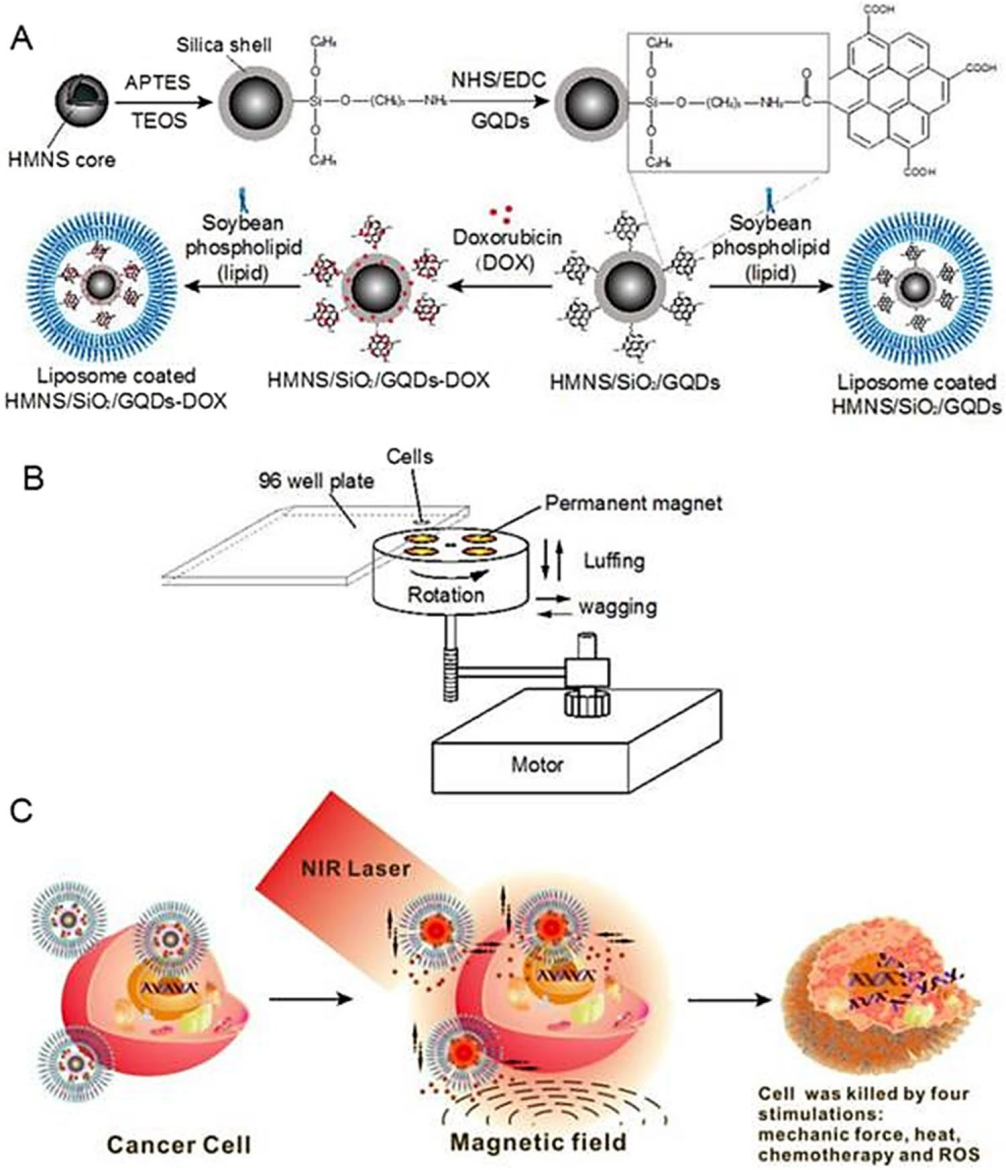
Schematic diagram of doxorubicin hydrochloride (DOX)-loaded nanocomposites [hollow magnetic nanosphere (HMNS)/SiO_2_/graphene quantum dots (GQDs)-DOX] that kill a cancer cell upon exposure to a dynamic magnetic field and near-infrared (NIR) laser irradiation. **A** The formation of liposome-coated HMNS/SiO_2_/GQDs-DOX nanocomposites. **B** The experimental setup of the dynamic magnetic field. Cells (96-well plate) are placed 1.4 cm above the magnets possessing a magnetic strength of 45.3 ± 0.5 mT and a rotation and swing of 2000 r/min. **C** The nanocomposites obtained exhibit multimodal therapy (mechanical force + heat + chemotherapy + reactive oxygen species) in cancer treatment when treated with an external magnetic field and NIR laser irradiation [119]

In a cooperative and multilateral manner, the multimodal system was able to destroy cancer cells via four distinct therapeutic mechanisms namely, magnetic field-mediated mechanical stimulation, photothermal damage, photodynamic toxicity, and chemotherapy. The innovative nanocomposites with combined mechanical, chemo and physical properties provide a different method for significantly enhancing the effectiveness of cancer therapy (see Fig. 10) [120, 121].

(iv) Polymeric nanoparticles

Polymeric nanoparticles (PNPs), mAb nanoparticles, extracellular vesicles (EVs) and metallic nanoparticles are broadly researched nanoparticles (NPs) for targeted delivery (Table 3). Colloidal macromolecules with submicron sizes of 10–1000 nm is referred to as PNPs. PNPs act as drug transporters for chemical medications, enabling their prolonged release to the intended malignant areas [122, 123]. A nanocapsule or nanosphere is created when drugs are insulated within or adhered to the surface of nanoparticles. Over time, nanoparticle components have undergone changes. Nanoparticles were first created using nonbiodegradable polymers such as polymethyl methacrylate (PMMA), polyacrylamide, polystyrene and polyacrylates [124, 125]. Polymeric nanoparticles produced by these materials need to be cleared up in a suitable manner to prevent toxicity and chronic inflammation. It is now possible to get these polymer-based nanoparticles destroyed, expelled, or physically removed from tissues without accumulating them to toxic levels. Biodegradable polymers have been created to optimise medication release kinetics, minimise toxicity, and boost biocompatibility. These polymers include chitosan, alginate, gelatin, albumin, poly(lactic acid), poly(lactic-co-glycolic acid), poly(amino acids) and poly(-caprolactone). These enhanced polymeric nanoparticles offer distinct benefits as a result of their characteristics and topologies. PNPs contribute to greater stability for unstable pharmacological compounds. In contrast to free drugs, PNPs have a higher loading capacity for chemical pharmaceuticals and offer optional distribution routes like oral and intravenous. Drugs’ capacity to resist degradation aids in reducing unintended toxicity to healthy tissues. For example, chemotherapy has used PNPs loaded with cisplatin such as dexamethasone or tocopheryl succinate, which inhibits cisplatin-induced ototoxicity [126].

**Table 3:** List of polymer nanoparticles for targeted delivery

Modification	Payload	Therapies involved	Target cancer model	Outcome	References
Exosome	Doxorubicin	Chemotherapy	Human breast cancer cells MDA-MB-231; mouse ovarian cancer cells; breast and ovarian cancer mouse models	Cytotoxicity of doxorubicin was enhanced and drug accumulation in mouse heart was avoided	[127]
PEG, transferrin modified NP	Nucleic acids	Nucleic-acid-based therapy	Human prostate cancer lines PC3 Chronic myelogenous leukaemia cells K562	Showed higher efficiency over untargeted particles when transfect K562 leukaemia cells	[128]
Tmab modified NP	Paclitaxel	Targeted therapy, chemotherapy	Human HER2-postive breast cancer cell lines: BT474, SK-BR-3; HER2-negative cell line: MDA-MB-231	Better treatment efficacy and lower cytotoxicity to human breast epithelial cell control were exhibited	[129]
Trithiol-terminated poly-methacrylic acid modified nanorods	Fe_2_P	SDT, PTT	Human cervix cancer cells HeLa; non-cancerous mouse fibroblast cells L929	The nanorod was biocompatible and showed ultrasound, photothermal synergistic therapeutic properties	[130]

## Nanoparticles delivery for cancer treatment

### Targeting cancer cells

The foremost aim of chemotherapeutic drugs for targeting cancer cells is to kill the cancer cells and to minimise the side effects [131]. For specific targeting of cancer cells using nanoparticles and medication delivery in cancer therapy, there are primarily two approaches used. Researchers are constantly working to improve various medications using nanocarriers to target cancer cells in a specific way.

**Fig. 11:**
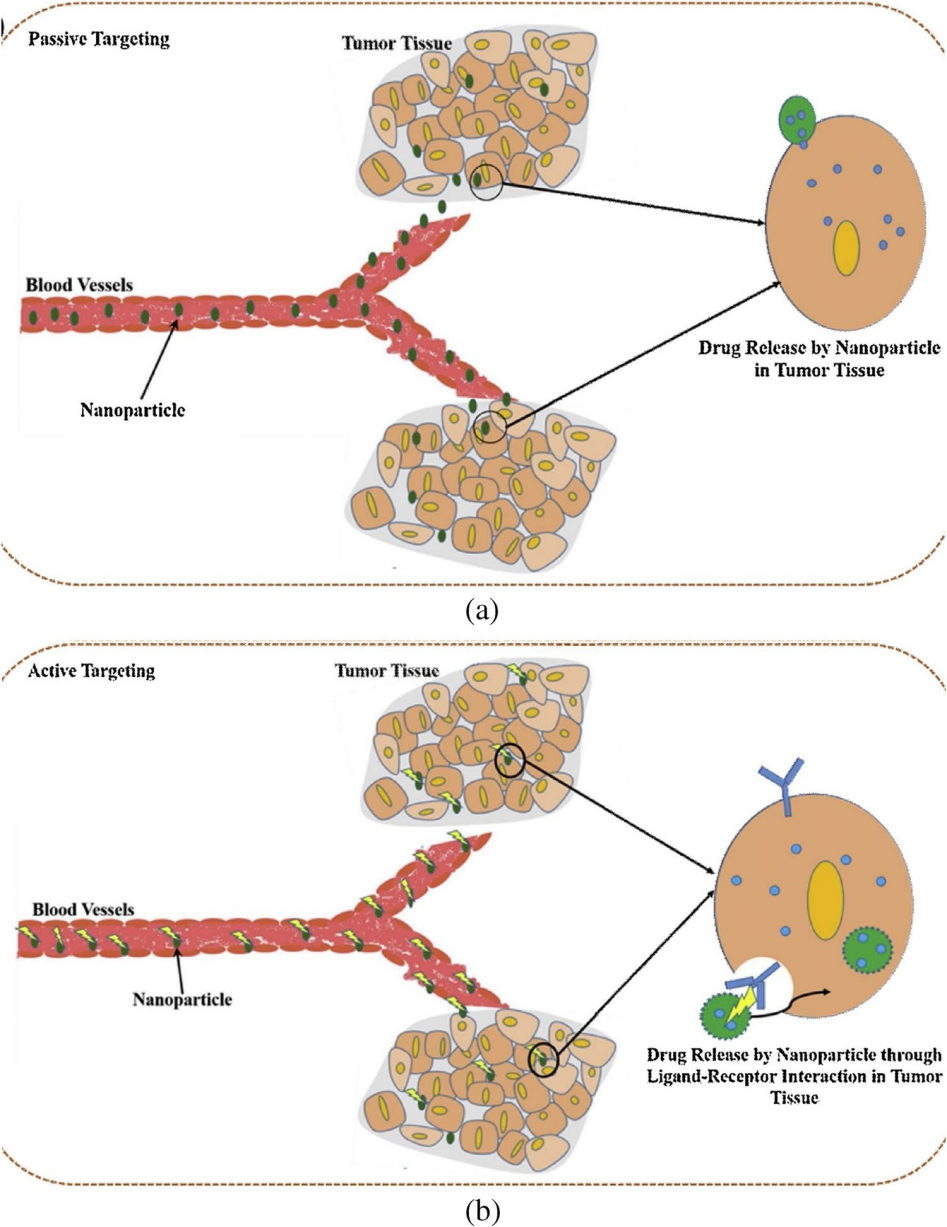
Mechanisms of tumour targeting by nanoparticles. **a** Passive targeting. **b** Active targeting [135]

In order to accumulate nanoparticle delivery systems including liposomes, polymeric-drug conjugates, micellar systems and polymeric NPs, passive targeting mostly relies on the physiological properties of the tumour. Rapidly developing tumours with enhanced vascular permeability and compromised lymphatic drainage frequently cause cancer and increase the permeability and retention (EPR) effect of nanosystems in that disease. Active cancer-targeting uses adding certain moieties to improve the delivery of nanoparticle systems to the tumour site [132]. Active targeting makes use of the highly expressed surface receptors on cancer cells by sustaining their engagement with the targeting ligands. In the earlier investigation on the active targeting of nanoparticles, several ligands composed of proteins (antibodies), nucleic acids, peptides or carbohydrates were employed [133]. These ligands can quickly attach to receptors that are expressed on cancer cells, mediating the binding and accumulation of NPs at the tumour site by receptor-mediated endocytosis and enabling the delivery of drugs for therapeutic action (Fig. 11). The two key criteria that determine the efficiency of active targeting are targeted specificity and deliverability. The nature and makeup of NPs directly affect the deliverability of nanoparticles [134]. The requirement that the desired NPs interact with the target antigen in close proximity makes developing active NP targeting a difficult task. Active targeting of nanoparticles for drug delivery enables effective encapsulation of NPs by target cells, and ongoing research is being done to demonstrate the effectiveness of drug delivery (Fig. 11) [135].

### Targeting the microenvironment

**Fig. 12:**
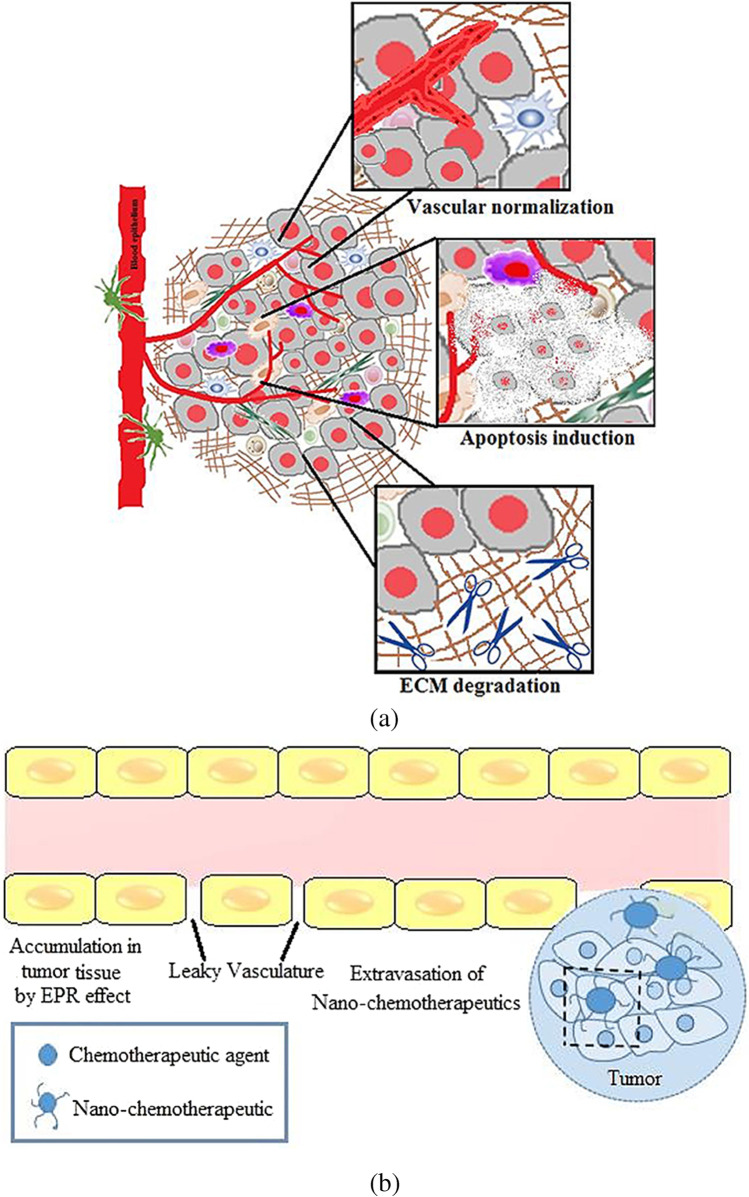
**a** Tumour microenvironment priming. **b** EPR effect in a tumour microenvironment [143]

Tumour microenvironment has been implicated in cancer growth and metastasis. With a better understanding of tumours, it is now known that they develop in a microenvironment that is very heterogeneous, complex, and made up of TAMs, CAFs, immune cells, and ECM components. Recent research shows that one key tactic in preventing cancer growth, invasion, and metastasis is altering the tumour microenvironment and its aberrant composition. With the development of nanotechnology in the drug delivery field, creative strategies to combat the cancer threat have emerged [136, 137]. However, it has emerged that the complexity of the tumour microenvironment has a significant, though debatable, impact on the control of nanochemotherapeutics’ higher tumoural penetration and, consequently, their biological effects [138]. To address this challenge, techniques have been developed employing nanotechnology that either target the tumour vasculature, change the stromal characteristics, or make use of the chemical microenvironment of the tumour to overcome acquired resistance caused by the tumour milieu. Therefore, by causing perturbations in the tumour microenvironment, nano-chemotherapeutics can change the way that drugs are delivered to tumours. Therefore, nanotechnology offers a flexible tool by permitting the delivery of either a solitary or combinations of chemotherapeutics together with numerous targeting ligands to specifically target overexpressed receptors or enzymes or a reductive environment, a characteristic of the tumour microenvironment [139, 140]. This strategy offers target specificity, resulting in effective therapy with little unintended negative side effects. Additionally, the development of a combination therapeutic and diagnostic method known as nanotheranostics is made possible by our improving understanding of how to target the tumour microenvironment utilising nanotools [141, 142]. Various approaches combining nano-chemotherapeutics with radiation and other related therapies will transform into a viable strategy for combating drug resistance since there are an increasing number of ongoing clinical trials on nanotherapy. Overall, it can be said that nano-chemotherapeutics do show promise in the early phases of cancer since these highly multifunctionalised nanocarriers enable delivery of chemotherapeutics either by utilising the tumour microenvironment or improved permeability and retention (EPR effect) as shown in Fig. 12 [143].

### NP and immunotherapy

Cancer immunotherapy, which activates body’s own immune system has become a viable method for treating a variety of cancers. By triggering a significant immune reaction against the tumour, this treatment not only destroys tumour cells but also stops them from getting back. High immune-mediated toxicity, ineffective and untargeted delivery of cancer antigens to immune cells, and off-target side effects are only a few of the daunting obstacles that therapeutic cancer immunotherapy must overcome. However, nanoparticle-mediated proposed system various ways to get beyond those restrictions and can thereby increase the effectiveness of immunotherapy. The primary problem in cancer immunotherapy is to deliver antigens for the subsequent development of an immune response [144]. To cause naive T-cell differentiation and activation as well as antigen presentation by the APCs for later CD8 + and CD4 + T cell activation, sufficient antigen and pretreatment are needed [145, 146]. APCs are immune cells that deliver antigens to the class I and class II MHC molecules on the surface of killer cells so that they can connect with T cell receptors. There are four main types of APCs in our immune system: DCs, B cells, macrophages and monocytes. The vascular endothelial cells, thymic epithelial cells, fibroblast, pancreatic cells and glial cells are further amateur APCs that work in certain circumstances [147]. In recent years, scientists have focused increasingly on developing immunostimulatory NPs that can be efficiently internalised by APCs. By doing so, they can transport cancer antigens and adjuvants to target cells selectively and stimuli-responsively, triggering an antigen-specific immune response [148–150]. These NPs enable the required immunostimulatory effects by allowing serum proteins to bind to their surface and form a corona that interacts with a variety of receptors [151]. In addition to targeted delivery, NPs could shield the cargo molecules from bioactivity loss during circulation and prevent adverse effects that are not intended [152].

Different types of cancer have responded well to cancer immunotherapy; nevertheless, a small percentage of individuals with particular tumour types show negative responses to immunotherapy, which restricts its widespread clinical use. According to the data available, hot tumours or tumours in the immune clearance stage, are virtually always cancers with several mutations, including melanoma, kidney cancer, NSCLC and hereditary rectal cancer. These tumours respond exceptionally well to immunotherapies, including PD1 inhibitors, which can greatly increase the time that cancer patients survive after diagnosis. Additionally, solid tumours like late-stage gastrointestinal tumours, triple-negative prostate cancer, malignant lymphoma, and some haematological cancers can easily induce tumour dormancy that encourages cancer spread and recurrence. These tumour types respond poorly to conventional treatment procedures; however, immunotherapy can eradicate these latent cancer cells by inducing an immune response. Other cancers, such as glioblastoma, pancreatic, prostate, and ovarian cancers, are mostly cold tumours at the stage of immune escape. The therapeutic impact of immunotherapy is diminished by this tumour immune escape effect. It should be emphasised that immunotherapy does not immediately reduce tumour size in people with advanced cancer. Immunotherapy as a post-operative adjuvant therapy can prevent recurrence and successfully prolong patients’ lives while maintaining their quality of life (Table 4) [153].

**Table 4:** Examples of nanotechnology-based formulations in clinical trials for cancer therapy

Type of nanomaterial	Product	Therapeutic agent	Indication	Status
Gold nanoparticle	Aurimmune	TNF	Solid tumour	Phase I
Anti-TfRscFv-decorated liposome: DOTAP, DOPE (1:1 molar ratio)	SGT-53	Human wild-type p53 DNA	Recurrent GBM, metastatic pancreatic cancer	Phase II
PEG-PEI-cholesterol lipopolymer	GEN-1	IL-12 plasmid	Epithelial ovarian, fallopian tube, and primary peritoneal cancers	Phase I/II
Targeted minicell	TargomiRs	miRNA mimic	Malignant pleural mesothelioma; nonsmall cell lung cancer	Phase I
Lipid NPs	ND-L02-s0201	siRNA	Advanced liver fibrosis	Completed Ib/II clinical trial
Lipid NPs	ARB-001467	siRNA	Hepatitis B	Completed clinical phase II studies
NBTXR3	Crystalline NPs	Hafnium oxide	Soft tissue sarcoma, liver cancer, prostate adenocarcinoma, head and neck cancer, NSCLC, rectal cancer	Phase II/III

### NP and drug resistance

**Fig. 13:**
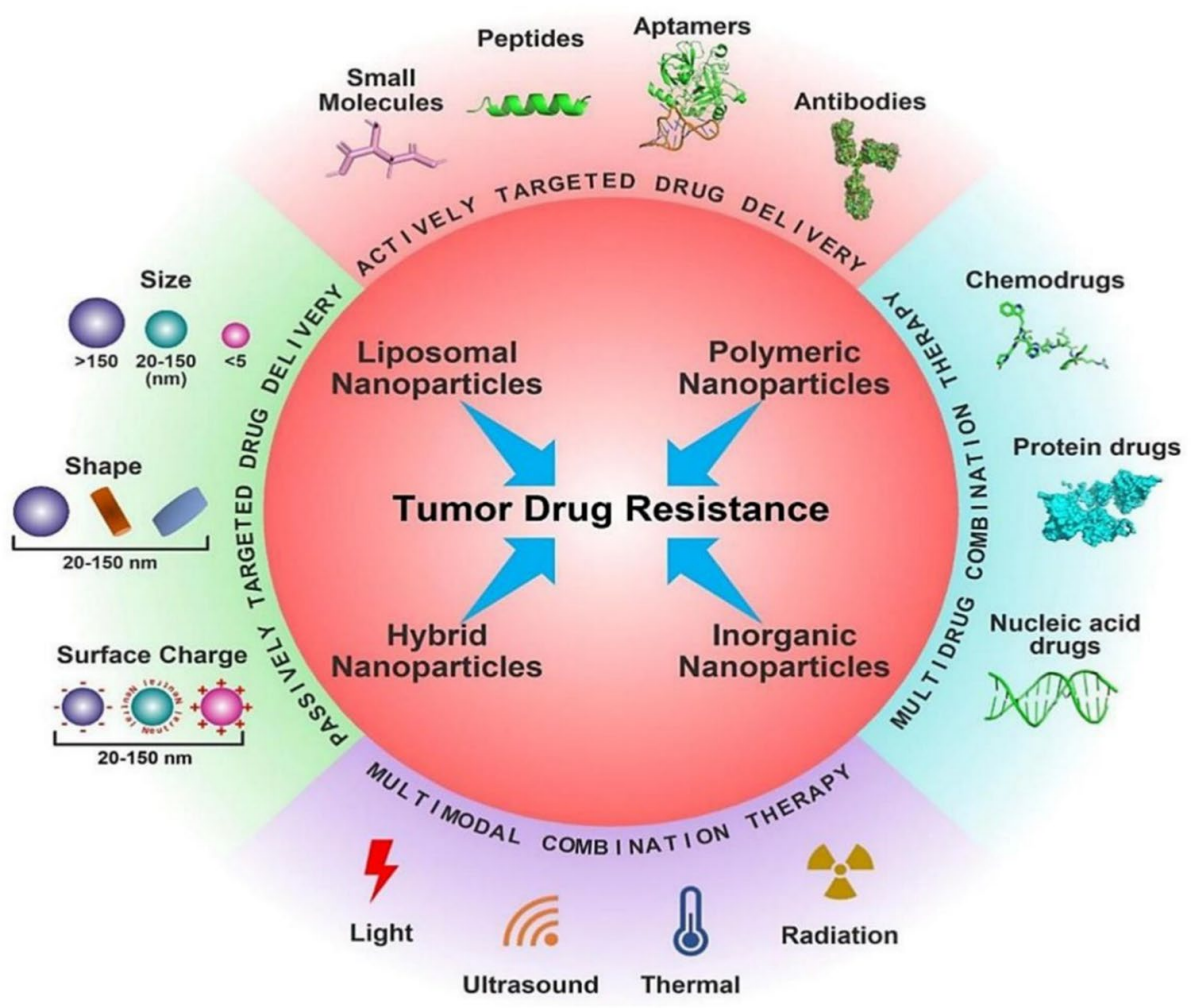
Different nanotherapeutic approaches for overcoming cancer drug resistance [158]

The most frequent reasons for chemotherapy failure, innate or acquired drug resistance, severely restricts the therapeutic results of chemotherapy. Recent developments in nanotechnology have offered substitute methods for addressing tumour medication resistance. Drug-loaded nanoparticles (NPs) are superior to free drug forms in a number of ways, including decreased cytotoxicity, prolonged blood circulation and greater tumour accumulation. However, due to the multiple pathophysiological hurdles present in the tumour microenvironment, such as intertumoral dispersion, penetration, intracellular trafficking, etc., nanoparticulate medicines have currently only minimally increased the overall survival rate in clinical studies. To increase the therapeutic effectiveness of nanomedicine, smart NPs with stimulus-adaptable physical and chemical characteristics have been developed in considerable detail. At the level of the tumour tissue, the drug resistance mechanism is highly intricate. Commonly regarded as the main drug resistance factors (Fig. 13), tumour heterogeneity, tumour microenvironment (TME), drug transporter and multidrug resistance, cancer stem cells (CSCs), epithelial-mesenchymal transition (EMT) and tumour metastasis all contributes to the off-target effect in the use of chemotherapy. Additionally, drug efflux caused by drug transporters compromises the delivery of cellular chemotherapeutics, resulting in low therapeutic doses [154–156]. Furthermore, the survival compensation effect may be strengthened by low pH, a hypoxic tumour microenvironment, and other anti-apoptotic chemicals [157]. Other resistant factors include gene mutations and genomic instability, epigenetic alterations including DNA methylation and protein acetylation, suppression of apoptotic signalling, and overexpression of anti-apoptotic molecules, in addition to the five medication resistance factors described [158].

The emergence of MDR has grown to be a significant issue in oncology and reduces the efficacy of chemotherapy in the management of many metastatic tumours. Resistance to numerous medications that are structurally and functionally different from the initial drug is highlighted by multidrug resistance. Increasing evidence suggests that resistance to cancer treatments is a difficult and complex process that needs serious attention right away and a thorough knowledge of the molecular principles [120]. According to the data currently available, medication resistance can be classified as intrinsic or extrinsic depending on the variables involved. Drug resistance can either be inherited or acquired, depending on the type of cancer. The doctors have a huge treatment challenge as a result of these two types of pharmaceutical resistance (inherited vs. acquired and extrinsic vs. intrinsic). Although cancer cells and their surroundings include resistance-mediating components which leads to resistance development. On the other hand, extrinsic or acquired drug resistance may appear while treating cancers that were previously sensitive to cytotoxic medications. Extrinsic resistance could arise through a variety of adaptive responses, such as the modification of signaling pathways, activation of alternative signaling pathways, and increased expression of the therapeutic target, and it would contribute to offset the therapeutic effects of previously used medications [159]. Additionally, the regulation and reprogramming of many metabolic and cellular physiological pathways, the tumour microenvironment, stemness, and cancer resistance are all impacted by the manipulation of signaling pathways.

Proinflammatory cytokines, chemokines and reactive oxygen species (ROS), among other elements, are crucial in the control of many signaling pathways. Overall, a variety of factors, causes, and mechanisms that are linked to drug resistance in various cancers (incorporating extrinsic and intrinsic resistance) include altering the tumour microenvironment, tumour heterogeneity brought on by cellular changes, reduced drug uptake, drug inactivation, altered drug targets, drug efflux, inhibiting cell death, changing the DNA repair process, epigenetics, inhibiting apoptotic pathways and autophagy and epithelium [160, 161]. Due to this, it is important to comprehend the phenomenon of cancer resistance as well as the fundamental signaling processes resulting from a variety of exogenous and endogenous elements in order to create future therapeutic interventions or combination therapies for diverse cancers. Table 5 shows a list of various factors that contribute to treatment resistance in cancers are shown.

**Table 5:** A representative list showing different mechanisms along with drugs, molecular targets and cancer type associated with cancer drug resistance

Resistance mechanism	Cytotoxic drugs	Type of cancer	Target	Reference
Microseminoprotein, prostate-associated (MSMP) gene upregulation	Vascular endothelial growth factor receptor 1/2/3 (VEGFR1/2/3) inhibitors	Ovarian cancer	Hypoxia, triggering mitogen-activated protein kinases (MAPK) signaling	[162]
Activated PDGFR	Histone deacetylase inhibitors, phosphatidylinositol 3-kinase, anti-VEGF drugs	Prostate cancer	Platelet-derived growth factor receptor (PDGFR)	[163]
Tumour heterogeneity	Tyrosine kinase inhibitors	Lung cancer	Epidermal growth factor receptor (EGFR) T790M mutation	[164]
Drug inactivation	Platinum drug	Lung cancer	Thiol glutathione	[165]
Reduced drug uptake	5-Fluorouracil (5-FU) and miR-21 inhibitor oligonucleotide (miR-21i)	Colon cancer	Micro-RNA-21 (miR-21)	[166]
DNA repair alternation	Platinum (carboplatin or cisplatin) and taxol (paclitaxel)	Ovarian cancer	DNA repair pathways	[167]
Inhibition in apoptotic pathways and autophagy	Epirubicin, tamoxifen, herceptin, and vinorelbine	Breast cancer	Autophagy	[168]
Epithelial to mesenchymal transition (EMT)	Wingless and Int-1 (Wnt) signaling inhibitors	Ovarian cancers	Wnt/β-catenin signaling pathway	[169]
Epithelial to mesenchymal transition (EMT)	Nivolumab	Urothelial cancer	EMT/stroma-related gene expression	[170]

## Future direction(s)

Artificial intelligence or AI refers to the simulation of human intelligence processes by machines, especially computer systems. In the last ten years, the field of AI has made much progress in vision, image/speech recognition and generation, planning, and decision-making. This has increased the role and importance of AI in all areas of healthcare most particularly in diagnosis, drug discovery and basic life science research.

However, the use of AI in healthcare is associated with challenges like lack of quality medical data and the gaps between the technical accuracy of AI tests and clinical usefulness. This has led to unreliable results many a times. Despite these challenges, AI is being increasingly used and trusted by healthcare professionals and industry. In the past years, AI has made unique contributions in anticancer drug development and treatment [171–173]. For instance, when it comes to formulating the most suited treatment for a patient, there is a possibility for doctors to choose unsuitable treatment where the patient is likely to miss on vital treatments opportunities which could result in delaying patient’s condition [174]. It is evident that AI has further capabilities of analysing, detecting, and processing information that humans are limited to, due to their level of knowledge.

AI has been integrated in various cancer fields including chemotherapy, radiotherapy and immunotherapy. The interaction between the drugs and the patients is the main focus of implementing AI in chemotherapy. The primary applications include chemotherapy drug use management, chemotherapy drug tolerance prediction, and chemotherapy programme optimisation [175–178]. AI can also be used in earlier detection of cancers. For example, the use of AI has enabled review and translation of mammograms faster with increased accuracy, reducing the need for unnecessary biopsies to detect breast cancers.

Additionally, the implementation of AI in radiotherapy assist radiologists to map out target areas or automatically plan radiation regimens for treatments as can be seen from the work from elsewhere [179–181]. Also, in the immunotherapy treatment, physicians uses AI to evaluate the treatment effect by adjusting the treatment plan for cancer patients [182–185].

AI can be used to add precision during surgery in determining cancer margins. Clear and adequate margins is a major determinant of completeness of surgery and cancer survival. AI and robotic surgery can help in accurate surgery which will preserve the function of organs without compromising on the oncological safety. Virtual reality simulation and AI can also help train future oncologists and surgeons. It is well recognised that AI can deliver critical information and insights that cannot be detected by human identification, thus personalising each cancer patient with suitable treatment [186–188]. Additionally, it is believed that AI can be a significant driver in human cancer research and treatment, by paving the way for the development of anticancer medication that could substantially accelerate the discover of new materials. It is also believed that AI will have a significant impact on the medical technologies in the upcoming years [174].

**Fig. 14:**
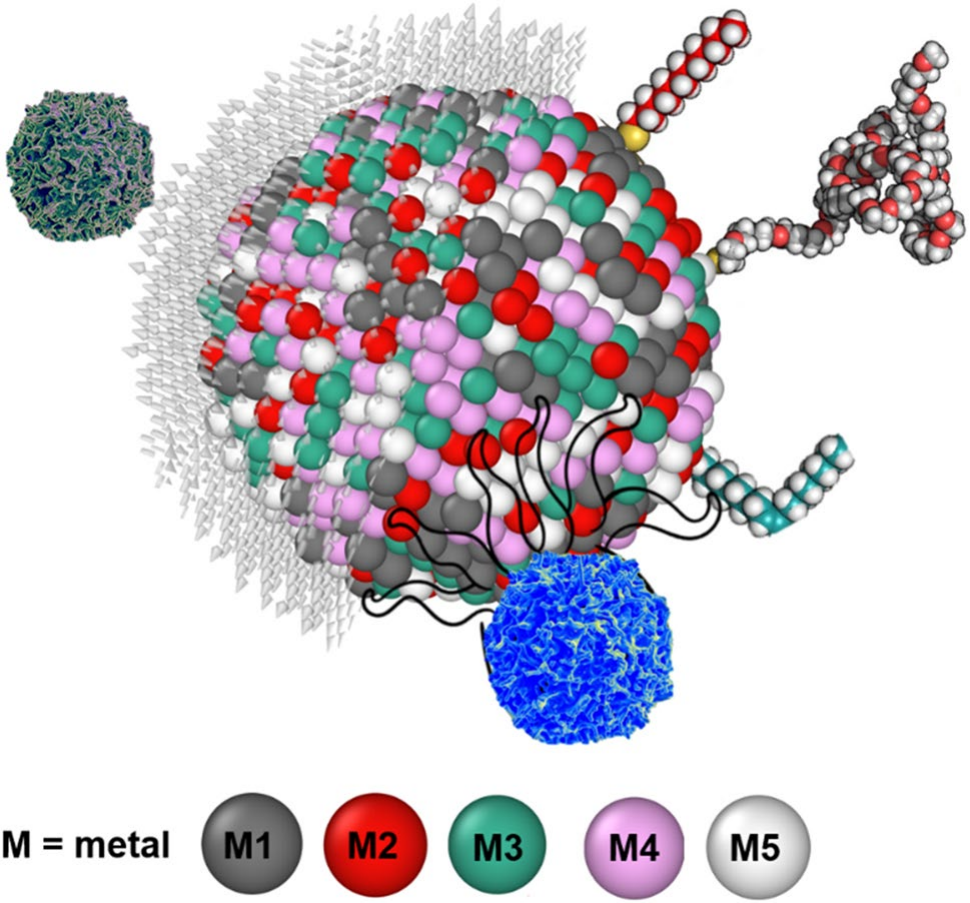
Interaction between HEA nanoparticle with a cancer cell. Distinctly different from simple metals, an HEA contain at least up to 5 chemical elements shown by M1, M2, M3, M4 and M5 and one out of these five elements is likely to be chemically affinitive to cancer cells making them vulnerable to succumb to HEA faster than single elemental NPs

While AI has the limits of finding out things from the data which is used to train the AI system, the radical innovation from AI cannot be expected. This type of innovation in cancer research will require fundamental basic sciences to develop and propose new ambitious measures. The authors of this review paper allude to one such possibility wherein the use of a new type of alloy nanoparticles can play a vital role in cancer treatment. We bring in the concept of high entropy alloy nanoparticles (HEAs) [189] which is a material system or an alloy containing five or more type of chemical elements arranged in a crystalline structure. These nanoparticles are known for their chemical homogeneity and could be promising for different kind of functionalisation because all metallic elements can reside over the surface of nanoparticles (Fig. 14), while pure element metallic nanoparticles have just one single element formulating the surface. Recently, HEA nanoparticles have shown prominence in the catalytic activity due to multiple binding sites over the surface [190, 191]. For example, the HEA nanoparticle surface atoms have different surroundings compared to their neighbour, which provide wide energy catalytic sites for analytes. As the paper alluded to the positive effects of nanoparticles (NPs), those benefits can best be realised by forcing a chemical reaction between an element contained in the NP and the cancer cell. However, not all cancer cell with initiate chemical reaction with an element. In that aspect, HEAs containing multi-elements offers higher probability for a reaction to initiate, and further accelerated by the presence of other surrounding (less chemically affinitive) particles (see Fig. 14). Additionally, the unique properties of NP obtained from their surface functionalisation can offer new abilities to study HEA nanoparticle interaction with biomatter for biosensing. HEAs gives wide space to manipulate or allow to bind with varying kinds of hetero-receptors. A major challenge with HEA nanoparticles is to fabricate free standing nanoparticles. In most cases, nanoparticle requires free standing (either powder form or suspended in solvent). Free standing nanoparticles are easy to be to be functionalised. Only few techniques such as carbothermal shocks method are available, where nanoparticles are supported over carbon nanofibre [192] and could not be separated from carbon nanofibre. Another method is cryomilling which is capable to prepare free standing nanoparticles in bulk quantity [193]. The best advantage of the HEA nanoparticles are the wide compositional space from the periodic table which means it is possible to mix different metallic elements to fabricate as many as 10^8^ different types of nanoparticle in equi-atomic or quasi-equi-atomic proportions [194]. However, only a few metallic nanoparticles have been used at the pre-clinical stage and it has been quite challenging to get FDA approval for advancement in this area. Consequently, the idea being proposed here has just only remained a hypothesis. As such, it is our mere perspective at this stage, that HEA NPs can contribute significantly to cancer sensing/treatment and further research in this area will help to validate this hypothesis.

## Concluding remarks

The field of magneto-mechanical anti-cancer therapies has exponentially grown for the last 5 years, paving the foundation for new therapies in oncology. The main efficacy demonstration was done *in vitro*, using highly heterogeneous magnetic-responsive nanoparticles and magnetic stimulation. Beside the inaugural thermal effect, the demonstration that mechanical stimulation can modulate the cell biology of cancer is further comforted by the recent demonstration of mechano-transduction pathways. These pathways as well as the connected physico-mechanic properties of the tissues are probably as important for the physiological tissue homeostasis than the classical molecular pathways governing the initiation and promotion of cancer. Only a few *in vivo* investigations have been done, contrasting with the number of *in vitro* studies. *In vivo* strategies are confronted by the bottleneck of tissue delivery that is very poor after intravenous injection. Intratumor delivery is a growing alternative in nanomedicine that has potential of becoming the first strategy, increasing the efficacy of local nanoparticle delivery as well as it decreases the potential systemic side effects. Anticipating from the beginning of these investigations, the biocompatibility of nanoparticle is mandatory. Moreover, it will also be mandatory to decipher the physical properties of the targeted tissue, such as stiffness and intra-tissular pressure that could modulate the efficacy of magneto-mechanical therapy. Several imaging methodologies have been developed for that such as ultrasound stiffness imaging. A mechanical dosimetry mapping of the tissue should provide the opportunity of a real mechanical therapy personalisation. Both *in vitro* and *in vivo* studies in the field of magneto-mechanical therapies of cancer pave the way for a real renewing of cancer therapies, responding to the therapeutical resistances observed in the field of chemotherapy and targeted molecular and cellular therapies. Translating physics and nanomagnetism will need a strong interdisciplinarity associating synergistically physician, biologists and physicists.

Through this review, we tried to demonstrate that the transport of NPs within the tumour microvasculature can be greatly influenced by various parameters such as size, shape, surface functionality, etc. The microcirculation of NPs and their subsequent internalisation by disease cells can be understood through computer simulation methods such as IMFEM and DPD. The important roles played by above parameters can be elucidated through these simulations. Thus, by combing the IMFEM with DPD simulations, the life journey of the NP-based drug carriers can be predicted through multiscale modelling approach. Through these multiscale simulations, fundamental mechanisms underpinning the NP-mediated drug delivery can be elucidated. These detailed physical insights can provide useful guidelines in the design of NPs. For instance, larger sized NPs were found to be able to migrate into the ‘cell-free layer’ whereas smaller sized NPs could be more efficiently taken up by the diseased cells. Based on these observations, a multistage delivery platform was designed by Ferrari and co-worker [195]. In the design of this platform, biodegradable and biocompatible mesoporous silicon particles were used to carry nanosized quantum dots or carbon nanotubes. During the microcirculation process, these mesoporous silicon particles can be more easily accumulated at the tumour sites due to the EPR and margination effects. Then, the NPs were gradually released, and they diffuse into the tumour cells. Through receptor mediated endocytosis and other pathways, these NPs can be internalised by tumour cells. Comparing with traditional design of NPs, this multistage platform has considered different physical mechanisms during the NP-mediated drug delivery process. Note that the traditional design of NPs relies on the slow and inefficient ‘Edisonian’ approaches. Such a process is very time-consuming and cost inefficient. According to the multiscale modelling approach, the design of NPs can be more easily achieved through the computer simulations. Soon, we hope that simulation-based design paradigms can guide experimental design of next-generation NPs, with enhanced active targeting, low toxicity and limited side effects.
